# Supplementary material for: Phenological Shifts in a Warming World Affect Physiology and Life History in a Damselfly
Source: Insects. 2022 Jul 12;13(7):622. doi: 10.3390/insects13070622 (PMC9318786; doi:10.3390/insects13070622)
Supplement: Supplementary file 1 [file insects-13-00622-s001.zip › insects-1782132-supplementary.pdf]

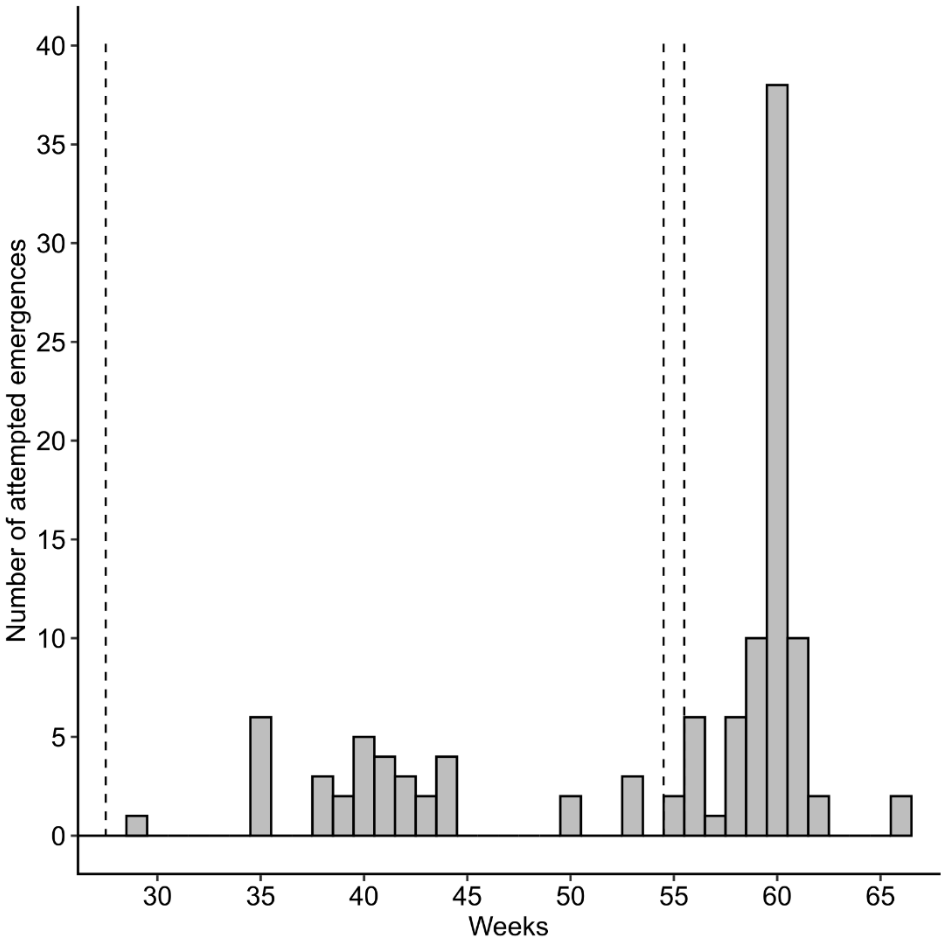

Figure S1. Number of attempted emergences per week in *Ischnura elegans*. No emergences happened during the first season (week 1-8), thus only second (week 28-55) and third (week 56-66) seasons were plotted. First dashed line mark the end of first winter. Second and third dashed lines represent beginning and end of second winter, respectively.

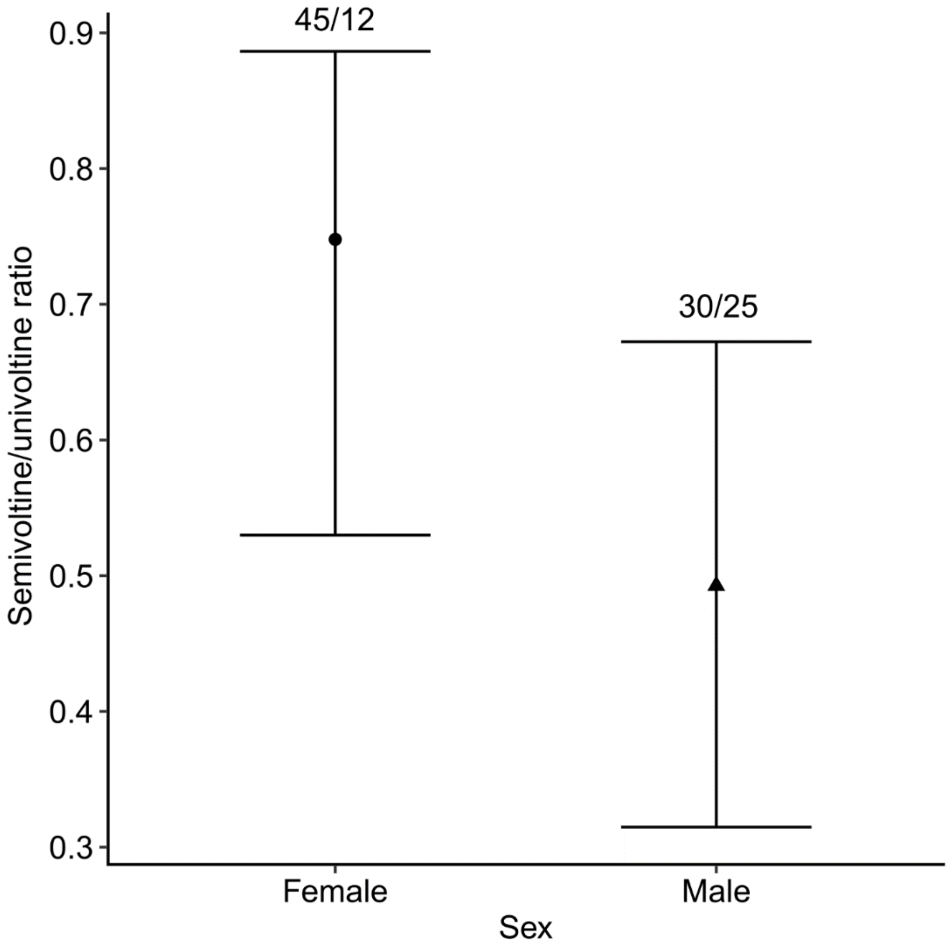

Figure S2. Semivoltine to univoltine ratio in emerging males and females. Error bars indicate estimated 95% CI. The numbers at the top of the error bars represent the number ratio of individuals within each group.

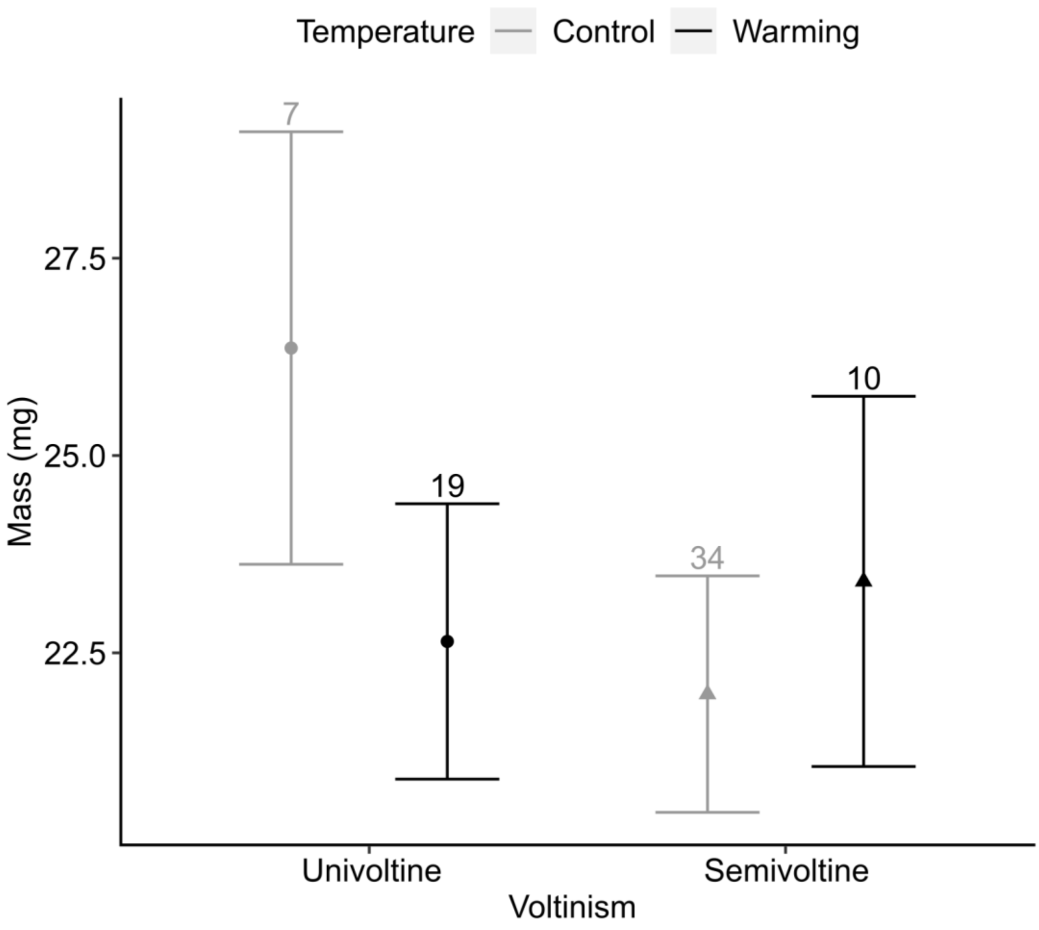

Figure S3. Mass at emergence in the control and warming temperature treatments, and across uni- and semivoltine individuals. Error bars indicate estimated 95% CI. The numbers at the top of the error bars represent the number of individuals within each group.

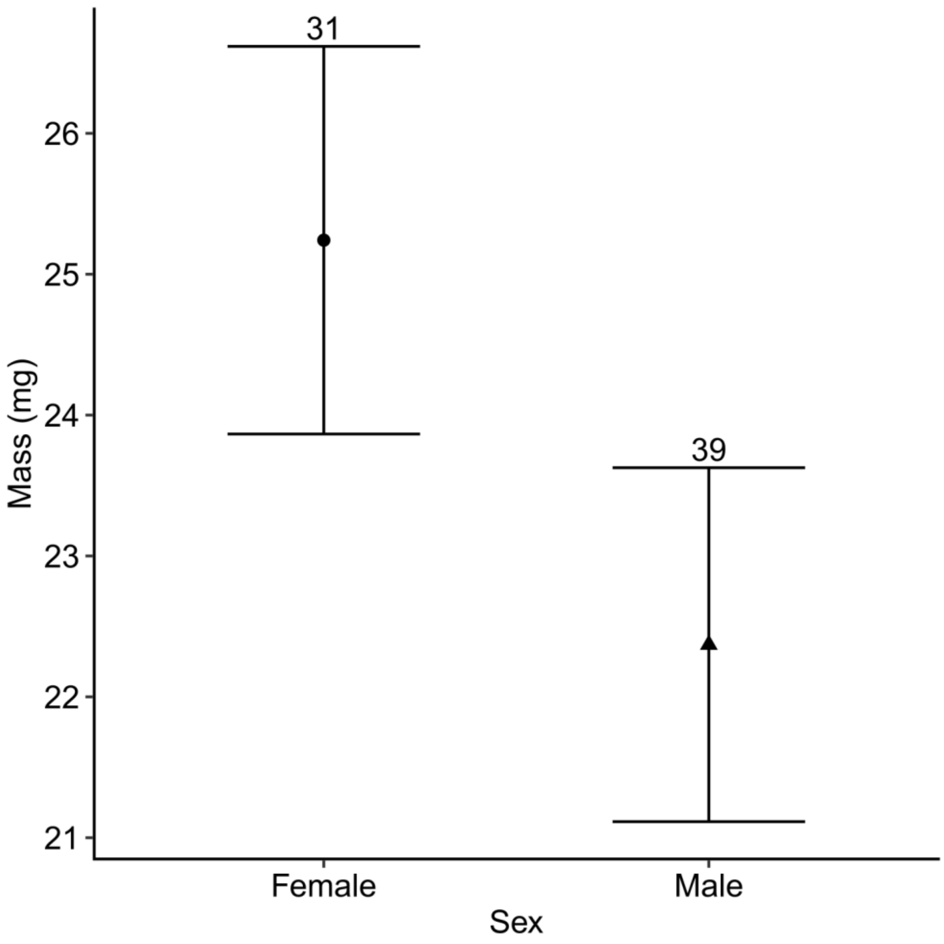

Figure S4. Female and male mass at emergence. Error bars indicate estimated 95% CI. The numbers at the top of the error bars represent the number of individuals within each group.

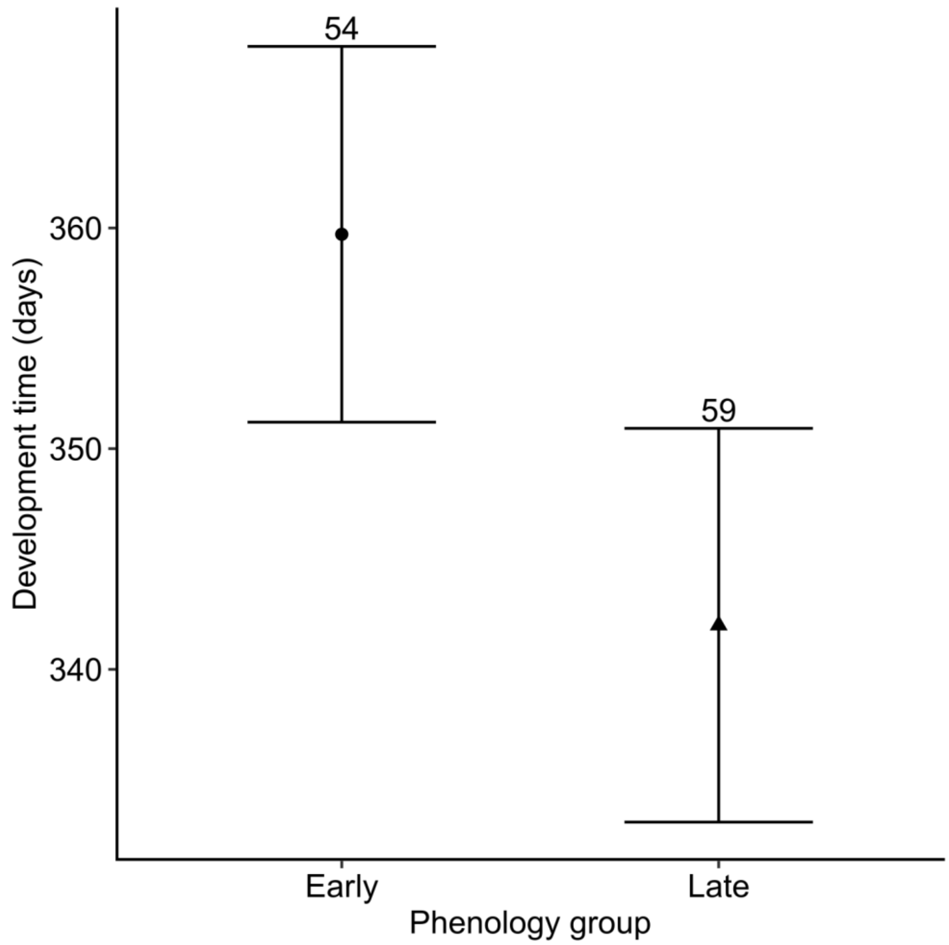

Figure S5. Development time for early and late hatchers. Error bars indicate estimated 95% CI. The numbers at the top of the error bars represent the number of individuals within each group.

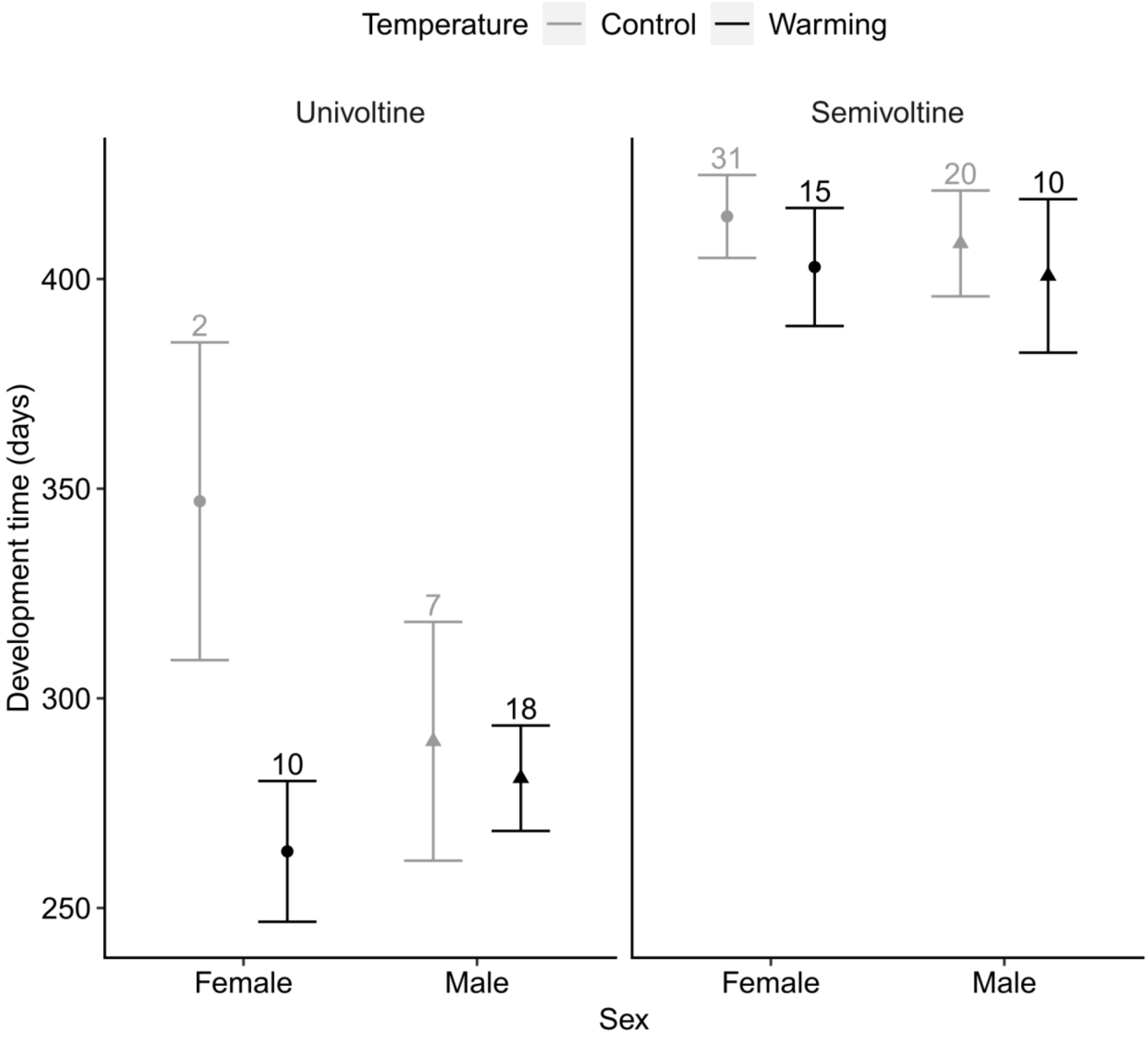

Figure S6. Development time in the control and warming temperature treatments for females and males, and across uni- and semivoltine individuals. Error bars indicate estimated 95% CI. The numbers at the top of the error bars represent the number of individuals within each group.

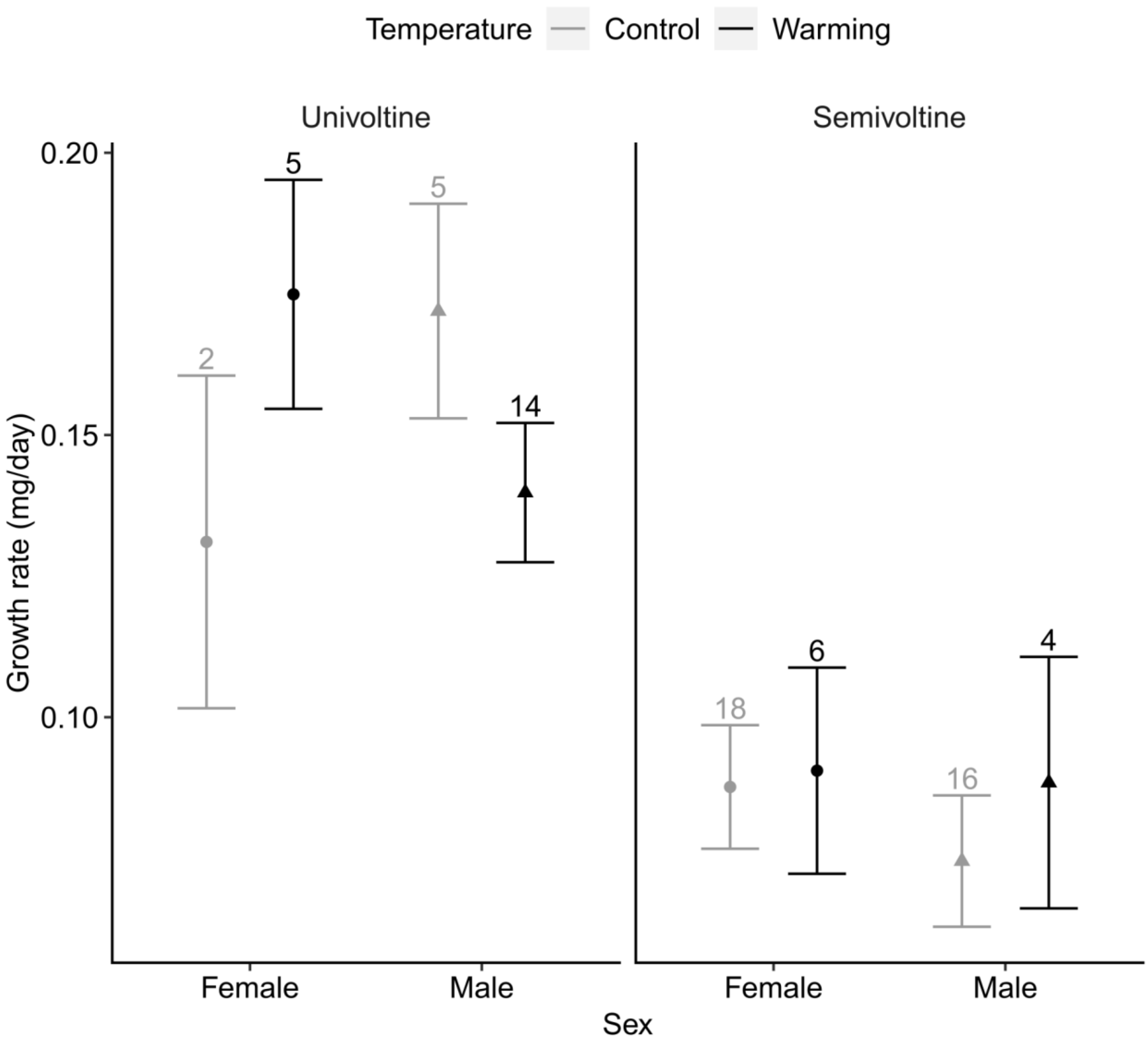

Figure S7. Growth rate in the control and warming temperature treatments for females and males, and across uni- and semivoltine individuals. Error bars indicate estimated 95% CI. The numbers at the top of the error bars represent the number of individuals within each group.

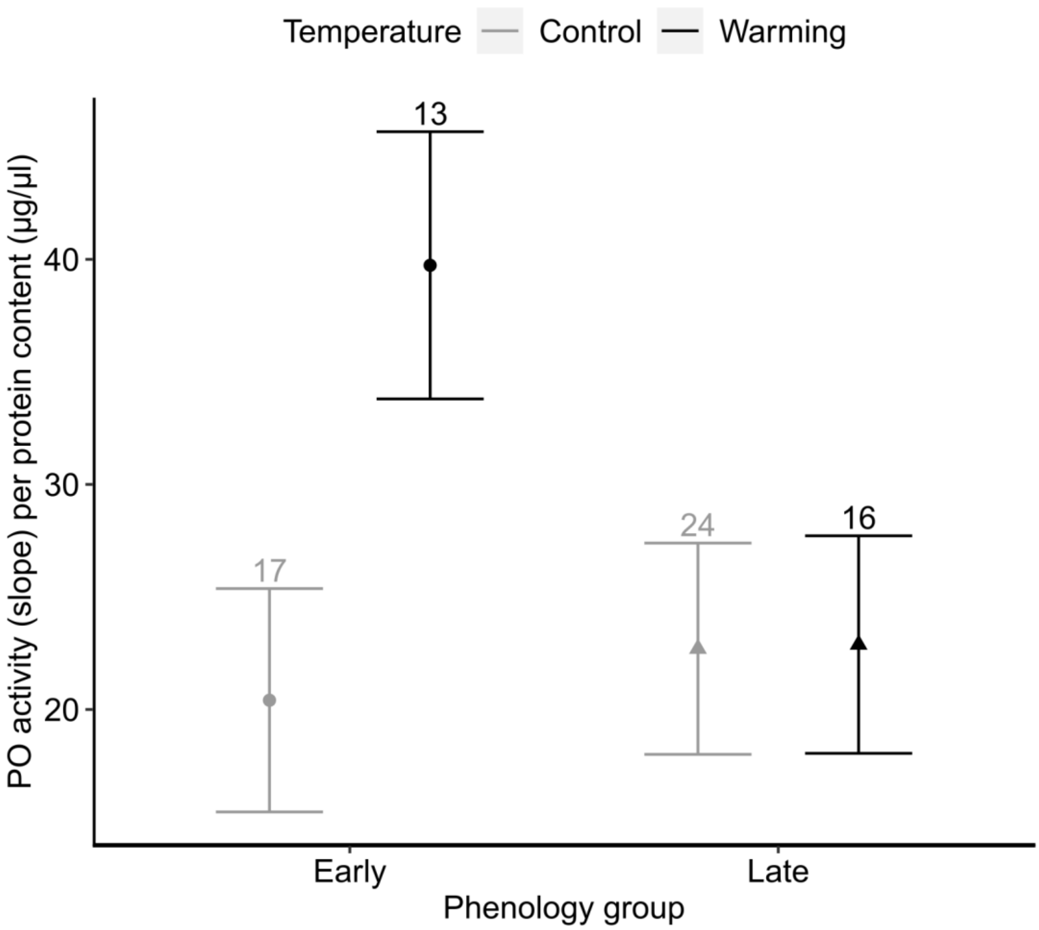

Figure S8. PO activity for early and late hatchers in the control and warming temperature treatments. Error bars indicate estimated 95% CI. The numbers at the top of the error bars represent the number of individuals within each group.

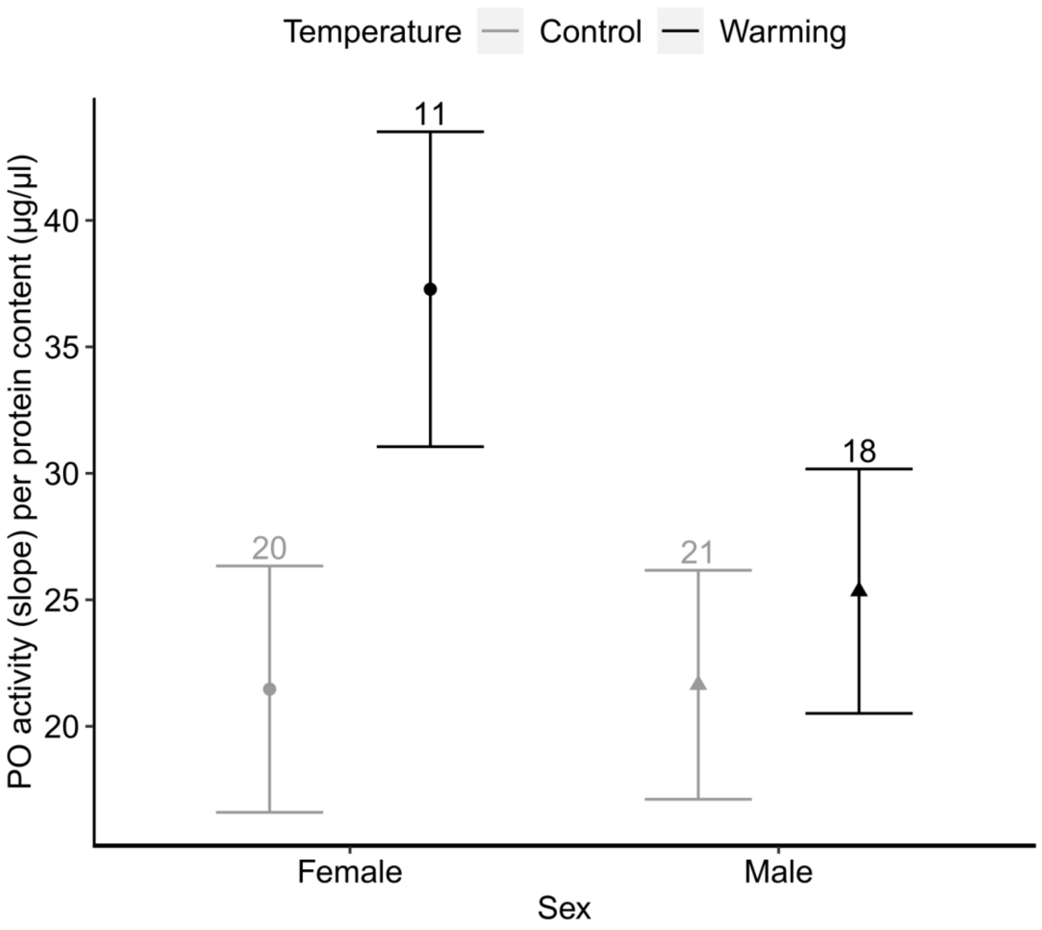

Figure S9. PO activity in the control and warming temperature treatments for females and males. Error bars indicate estimated 95% CI. The numbers at the top of the error bars represent the number of individuals within each group.

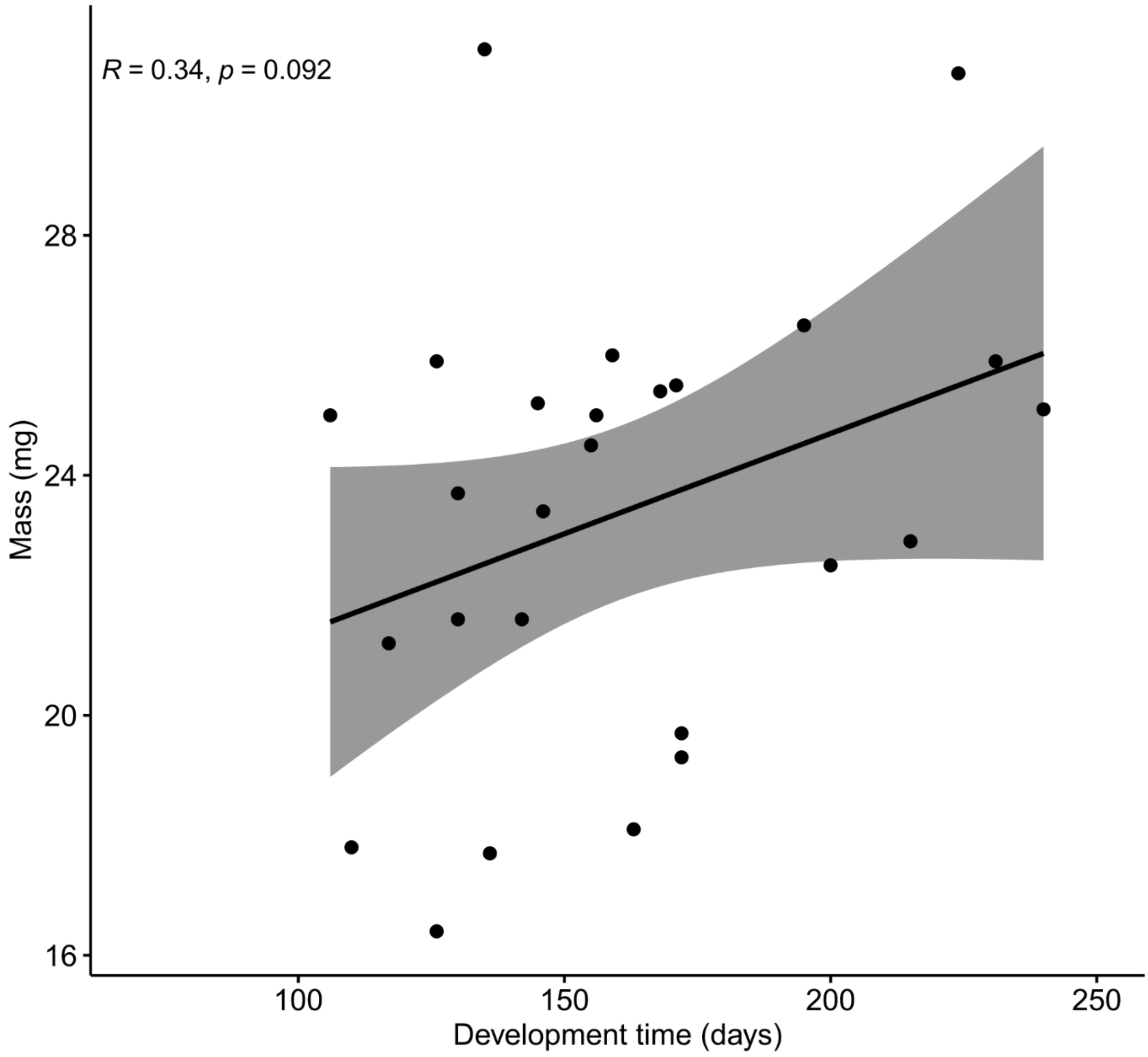

Figure S10. Correlation between mass at emergence and development time. Grey area represents estimated 95% CI.

Table S1. Survival until emergence and emergence success of *Ischnura elegans* across growth seasons. Season 1 lasted between 16 July 2019 and 05 Sept 2019 (equivalent of experimental 4 Aug 2019 - 23 Dec 2019). Season 2 lasted between 24 Jan 2020 and 24 July 2020 or (equivalent of experimental 8 May 2020 – 06 Nov 2020). Season 3 lasted between 7 Aug 2020 and 13 Nov 2020 or (equivalent of experimental 15 May 2020 – 6 Nov 2020). Only individuals which emerged successfully and survived to wet mass measurement (i.e., until next day after emergence) were used for mass, growth rate and PO analysis.

Seasons	1	2	3
Survival until emergence	0	37	76
Emergence with success	0	35	57
